# A prospective cohort study assessing the clinical utility of the Cottle maneuver in nasal septal surgery

**DOI:** 10.1186/s40463-018-0292-9

**Published:** 2018-07-11

**Authors:** James P. Bonaparte, Ross Campbell

**Affiliations:** 1 Department of Otolaryngology – Head and Neck Surgery Senior Clinical Investigator, The Ottawa Hospital Research Institute, University of Ottawa, 1919 Riverside Drive, Suite 308, Ottawa, Ontario K1H 7W9 Canada; 2 Department of Otolaryngology – Head and Neck Surgery, The University of Ottawa, Ottawa, Canada

**Keywords:** Septoplasty, Nasal obstruction, Nasal valve collapse, Cottle maneuver

## Abstract

**Background:**

A nasal septal deviation can have a significant detrimental effect on a patient’s quality of life. Nasal valve collapse (NVC) often co-exists with a septal deviation. The Cottle maneuver is one of the most common methods to diagnose NVC; however, no study has assessed the efficacy of this physical exam finding. This study tests the hypothesis that patients with nasal obstruction due to a septal deviation with a negative pre-operative Cottle maneuver will demonstrate a greater improvement in their Nasal Obstruction Symptom Evaluation (NOSE) score, compared to patients who demonstrate a positive pre-operative Cottle maneuver, when assessed at 12 months following a septoplasty with turbinate diathermy.

**Methods:**

This was a prospective Cohort Study. The population was 141 patients with nasal obstruction due to a septal deviation with or without nasal valve collapse, excluding patients with bilateral complete nasal valve collapse. Patients were placed in cohorts according to the results of the Cottle maneuver (positive or negative). A NOSE questionnaire was administered at baseline and 12-months after a septoplasty with turbinate diathermy. Non-adjusted NOSE scores were used (score out of 20). An ANOVA was used to compare if there was a difference in outcomes between patient cohorts.

**Results:**

One hundred and forty-one patients completed 12-month follow-up with 71.5% of patients demonstrating a positive Cottle maneuver at baseline. The mean (95% C.I.) difference in NOSE score at 12 months between patients with a positive Cottle versus a negative Cottle was 0.18 (− 1.6 to 1.92; *p* = 0.38).

**Conclusion:**

In a univariate, single surgeon study, a positive Cottle Maneuver does not appear to influence outcomes in the described patient population compared to those with a negative Cottle Maneuver when undergoing a septoplasty.

## Background

Nasal obstruction is the most common sinonasal complaint with which patients present to an otolaryngologist [1–3]. A nasal septal deviation, a common cause of nasal obstruction, can have a significant detrimental effect on a patient’s quality of life [4]. Nasal valve collapse (NVC) often co-exists with a septal deviation [5–10]. Although physicians have studied objective measures to diagnose NVC, the vast majority rely on physical exam findings [5, 6, 11]. A systematic review by Speilmann et al. [6] identified 43 papers assessing the treatment of nasal valve collapse. Of those, 24 papers utilized the Cottle maneuver to diagnose nasal valve collapse, 11 did not specify the method of diagnosis, while only one study utilized objective measures, specifically rhinomanometry. Of the studies that employed the Cottle maneuver, five utilized the Cottle maneuver as a single variable, while the remainder used a combination of the Cottle maneuver and a subjective assessment of intra-nasal support for their formal diagnosis of nasal valve collapse. Needless to say, the Cottle maneuver is a common component of the nasal examination [12, 13] and a common method to diagnose NVC.

To conduct the Cottle maneuver, the patient is required to inspire while the physician applies tension on the skin lateral to the nasolabial fold, thereby increasing nasal wall tension and widening of the nasal valve. In patients who have narrow or collapsing nasal valves, this maneuver improves nasal airflow, which constitutes a positive test. To many physicians, a positive test suggests that a functional rhinoplasty to specifically address the nasal valve may be necessary [6]. Indeed, in a clinical consensus statement published by the American Academy of Otolaryngology – Head and Neck Surgery (AAO-HNS), [13] the authors researched a consensus regarding the utility of certain physical exam findings in diagnosing NVC. These include: the subjective improvement in nasal airflow during a Cottle maneuver, the visible inspiratory collapse of the nasal wall and/or alar rim during inspiration, and the increased nasal obstruction during deep inspiration. Audible improvement in nasal airflow along with subjective improvement during the Cottle maneuver reached consensus; however, audible improvement alone did not. Interestingly, there was a consensus that there is no gold standard test to diagnose NVC.

As mentioned previously, results of a systematic review [6] noted that the Cottle maneuver is the most common method used to diagnose clinically relevant NVC that requires surgical repair. Of the studies reviewed, 55% of papers reviewed relied on the Cottle maneuver alone or in combination with a physical exam as the definition of clinically relevant NVC. Despite the widespread acceptance of the Cottle maneuver as a physical examination test to diagnose and define NVC, it has never been validated, nor has it been confirmed that all patients with a positive Cottle maneuver require repair of the nasal valve.

Based on our review, the majority of studies that assessed the effectiveness of a septoplasty in treating nasal obstruction secondary to a septal deviation have used the evidence of NVC as an exclusion criterion [14–21]. A number of other studies assessing the outcomes of septoplasty indicated that other causes of nasal obstruction were excluded, but made no specific reference to nasal valve collapse [14, 22–26]. We found only one study that assessed septoplasty outcomes in patients with a septal deviation along with evidence of NVC [7]. However, given that the nasal septum and inferior turbinates themselves constitute boundaries of the internal nasal valve, [5, 6, 11, 13, 27–32] a septoplasty with a reduction in the anterior edge of the inferior turbinate will theoretically have an effect on the internal nasal valve. Garcia et al. [33] assessed nasal resistance due to a septal deviation at different points of the nasal cavity. The authors noted that a septal deviation located at the level of the nasal valve (within 3 cm of the nasal opening) resulted in an increase in nasal resistance by 124%, while deviations in other areas of the nasal cavity increased resistance by no more than 30%. This increase in resistance could in itself alter the biomechanics of nasal airflow and thereby alter transnasal pressure and thereby result in alar or valve collapse.

Schalek and Hahn (2011) [7] noted that, in patients with an anterior septal deviation along with contralateral nasal valve collapse, a septoplasty led to resolution of both the nasal obstruction and nasal valve collapse. The authors noted that 91% of patients demonstrated an improvement on the side with nasal valve collapse. This study was limited by its small sample size of 12 patients, and the lack of a validated outcome measure. The clinical consensus statement published by Rhee et al. [13] noted that there was a strong consensus that procedures targeted to support the lateral nasal wall/alar rim are distinct entities from a septoplasty. However, there was moderate to strong agreement that, “in some cases” a septoplasty can treat NVC without other nasal wall procedures. The authors note that surgical procedures targeting the nasal wall are indicated when septal and/or turbinate surgery is not sufficient. Apart from the previously discussed study, there is little or no research specifically assessing the role of a septoplasty in patients with NVC. Therefore, the effectiveness of a septoplasty with inferior turbinate treatment alone in treating nasal obstruction secondary to a septal deviation with co-existing NVC has not been adequately studied.

Given the recommendations for diagnosing NVC as well as the frequency in which studies utilize the Cottle maneuver to diagnose it, one should question whether the test is clinically useful in patients with a septal deviation. If the Cottle maneuver accurately diagnoses NVC, and by extension, patients that also require surgery of the nasal valve, one can make the assumption that patients with a septal deviation and a positive Cottle maneuver may require specific treatment of the nasal valve. This is turn would suggest, that if patients require nasal valve surgery in addition to a septoplasty, not performing this required surgery might result in poorer outcomes compared to patients with a septal deviation who require only a septoplasty with or without inferior turbinate diathermy. However, this too has not been adequately studied.

The primary objective of this study was to test the hypothesis that patients with nasal obstruction due to a septal deviation who have a negative pre-operative Cottle maneuver will demonstrate a greater improvement in their Nasal Obstruction Symptom Evaluation (NOSE) score, compared to patients who have a positive pre-operative Cottle maneuver, when assessed at 12 months following a septoplasty with turbinate diathermy [15].

The secondary objective was to test the hypothesis that the odds of failure of a septoplasty, as defined by a published patient centered outcome [34] using the NOSE score, would be higher in patients with a positive pre-operative Cottle maneuver versus a negative Cottle maneuver.

## Methods

### Study design

This study was approved by our institutional ethics review board (20140735-01H). This was a prospective cohort study, consisting of two groups of patients with nasal obstruction. All patients were diagnosed with a septal deviation with or without visible evidence of NVC. Patients underwent a thorough standard pre-operative clinical evaluation of the nasal airway, including administration of the NOSE score. Patients were then placed in groups depending on the result of the Cottle maneuver, either positive or negative.

### Population

All adult patients over the age of 18 years old, referred to the otolaryngology clinic of the senior author (JB) between Nov 1, 2014 and March 1, 2017 with nasal obstruction with a septal deviation were asked to enroll in the study. All patients had a minimum of a one-month trial on a topical intranasal corticosteroid prior to enrollment in the study.

Patients with bilateral partial NVC or unilateral complete NVC in addition to a septal deviation, either unilateral or bilateral (Grade 0–2 OVCS: Ottawa Valve Collapse Scale), [35] were included in the study. Partial collapse was defined as collapse of the internal and/or external valve during inspiration with the maintenance of nasal airway airflow; complete collapse was defined as total collapse of external nasal valve with the nasal ala contacting the caudal septum during inspiration, thereby completely occluding nasal airflow. Patients with complete bilateral collapse of the external nasal valve during inspiration were considered to have severe NVC and were excluded (Grade 3 OVCS). Patients were also excluded from the study if they previously had nasal structural surgery, static narrowing of the alar rim or external nasal valve (ie. a caudal septal deviation along the columellar edge, wide columella, statically collapsed alar rim), co-existing traumatic deviation of the nasal bones, allergic rhinitis, chronic rhinosinusitis with or without nasal polyposis, a neoplastic or autoimmune process.

### Assessment of nasal airway

All patients had a thorough otolaryngological physical examination. Specifically, the external structure of the nose was assessed, and any deviation of the bony nasal pyramid or other deformities was documented. Visual inspection for collapse of the internal and/or nasal valves with both normal and deep inspiration was performed, and the presence or absence, laterally and severity of observed nasal collapse was recorded. A nasal speculum was used to perform anterior rhinoscopy and finding of a septal deviation and/or inferior turbinate hypertrophy were documented. Nasal decongestion was not utilized as all patients had a minimum of 1 month trial of topical nasal corticosteroids prior to inclusion. Flexible nasolaryngoscopy was performed in all patients to rule out non-septal causes of nasal obstruction.

All patients had the Cottle maneuver performed pre-operatively by the primary author (JB) as part of a general nasal examination. The examiner instructed the patient to breathe to breath in deeply through his or her nose two times. The first with no intervention, and the second time with the examiners’ thumbs placed on the patients’ cheeks, applying firm lateral pressure to stent open the nasal valves. A patient was defined as having a positive Cottle maneuver if he/she indicated his/her breathing improved compared to breathing without the Cottle maneuver. Finally, a baseline NOSE score was obtained for each patient.

### Intervention

All patients had a septoplasty with bilateral inferior turbinate diathermy performed by the senior author in Ottawa, Ontario, Canada. The surgical approach was similar for all patients. A Killian incision, placed approximately 0.3–0.5 cm from the edge of the columella on the left side, was performed for all patients. A unilateral mucoperichondrial flap was raised on the left side. The deviated portion of the septum as well as the maxillary crest, if deviated, was removed. The surgery was individualized in accordance with the patient’s individual anatomy and sites of obstruction. The L-strut of the septum was not altered according to standard practice. The mucoperichondrial flap was then closed using a 4–0 gut quilting suture followed by 4–0 gut closure of the Killian incision. No septal splints or packing were used in any patient [36]. The anterior edges of the inferior turbinates were reduced using needle-tip electrocautery set on 15 coagulation in a submucosal fashion. The turbinates were then lateralized by out-fracturing the bone. Follow-up for patients occurred between one and 2 weeks post-operatively for initial assessment, and again at 1 month, 6 months and 12 months.

### Outcome measure

The primary outcome measure utilized for the study was the NOSE [15, 20] score at 12 months post-operatively. The relative change in NOSE score, defined as the percentage change as a function of baseline score, was not used as this would convert normally distributed data into non-normal distribution [37]. Instead, the NOSE score at 12 months was used as the primary outcome and the baseline NOSE score [22] was used as a covariate to correct for baseline differences in symptom severity in an ANOVA [37].

A secondary outcome measure, surgical failure, was defined as an improvement in the NOSE score of 40% or less at 12 months; this value has recently been shown to be the minimal important difference for patients, in a study of patient-defined outcomes following nasal airway surgery [34]. Using this definition, we were able to dichotomize outcomes into treatment success or treatment failure [34].

A physical exam was performed to document any complications at 12 months. In patients who did not meet the definition of a successful surgery, we attempted to identify the reason for failure. To identify dynamic internal or external nasal valve collapse post-operatively, the Modified Cottle maneuver [38] was used. Static collapse was assessed subjectively if patients appeared to have a narrow valve that did not improve with the Modified Cottle maneuver. Caudal septal deviations were defined as a septal deviation occurring within the area of the external nasal valve.

### Statistical analysis

A pilot test of 25 patients without complaints of nasal obstruction resulted in an average NOSE score of 2.26 with a standard deviation of 3.06. while those with nasal obstruction had a mean score of 15.68 with a standard deviation of 2.96 [39]. Assuming a power of 95% and a *p*-value of 0.05, and significance difference between groups defined as 3 with a standard deviation of 3.5, a minimum of 37 patients per group would be required for the study. With this study, we aimed to enroll a minimum of 40 patients per group to ensure an analysis of covariates and subgroup analysis could be performed.

All summary data was presented as mean (standard deviation). An Anderson-Darling test was used to assess the NOSE score for a normal distribution. A general linear model ANOVA was used to compare patients with and without a positive Cottle maneuver. The outcome measure was NOSE score at 12 months. The pre-operative Cottle maneuver result (positive or negative) was used as the categorical variable. Gender was included as a potential variable. Age and baseline pre-operative baseline NOSE score were used as covariates. Statistical significance was defined as a *p* < 0.05.

To assess our secondary objective, a logistic binary regression was used to assess whether a positive Cottle maneuver increased the odds of a failure of a septoplasty. The definition of failure was based on a patient centered outcome [34]. Specifically, if a patient did not improve their NOSE score by 40% or more, patients were considered to have failed surgical intervention.

A chi-square test was used to determine if there was a relationship between patients with a positive Cottle maneuver and visible evidence of NVC.

## Results

**Fig. 1:**
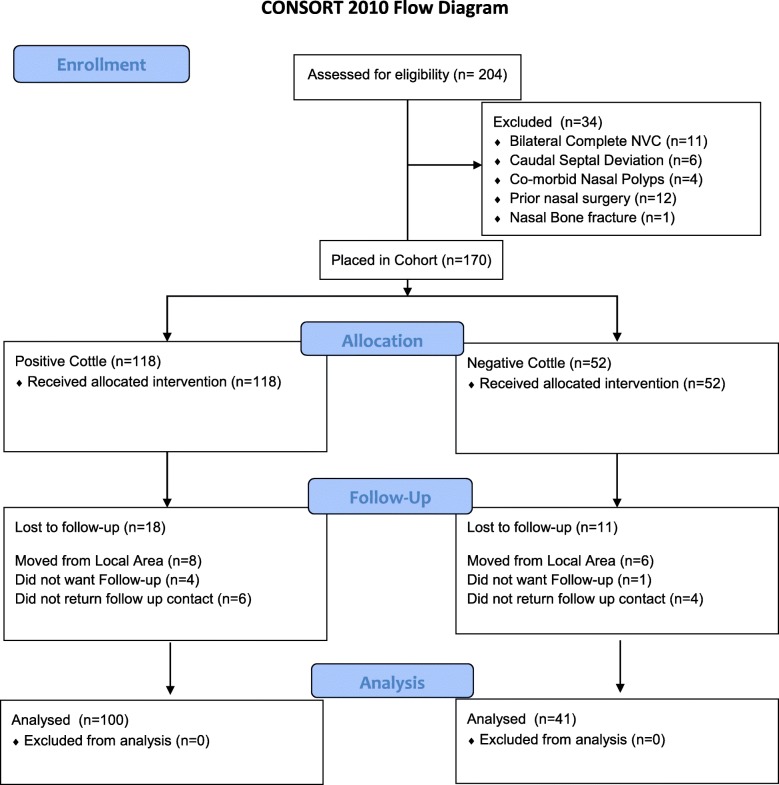
CONSORT 2010 Flow Diagram

A total of 181 patients were screened for inclusion (Fig. 1). A total of 170 patients provided baseline data and completed the surgical treatment. One hundred and forty-one (141) patients completed the 12-month follow-up data collection, corresponding to a drop-out rate of 17%,; 21.1% in negative Cottle cohort and 15.2% in positive Cottle cohort.

The mean (standard deviation) age of patients who completed follow-up was 41.3 (13.4); 28.5% of patients were female. After a baseline screening exam, 67.4% of subjects had a positive Cottle maneuver. Summary data for all patients are presented in Table 1. The NOSE data at 12 months did not differ from a normal distribution (*p* = 0.23).

**Table 1:** Summary of demographics and baseline outcome measures. Although scores for the change in NOSE and the percentage change in NOSE relative to baseline are presented, this data has not been controlled for the covariates included in the ANOVA model

Variable		All Patients	Positive Cottle	Negative Cottle	*p*-value
Patient Count	N	141	95	46	n/a
Age	Mean	41.3	40.4	43.4	0.17^a^
	SD	13.4	12	16.3	
Gender	% Female	28.5	23.1	41.9	0.007^b^
Baseline NOSE	Mean	13.3	13.1	13.7	0.43^a^
	SD	4.06	4.1	3.9	
12 Month NOSE	Mean	4.1	4.2	4.2	0.57^a^
	SD	4.6	4.6	4.5	
Change in NOSE	Mean	9.1	8.9	9.5	0.78^a^
	SD	5.7	5.8	5.6	
% Change in NOSE	Mean	67.5	66.8	68.8	0.81^a^
	SD	35.8	36.9	33.5	
Surgical Failure	n	14	9	5	0.99^b^
	% of Total	9.9	9.5	10.5	

*N* number of patients, *SD* standard deviation, *NOSE* nasal obstruction symptom index, *n/a* not applicable

^a^two-sample t-test, ^b^ Chi-square test

Of those with a negative Cottle maneuver, 56.5% had no evidence of visible valve collapse while 43.5% had visible evidence of valve collapse on exam. For those with a positive Cottle maneuver, 41.0% had no visible evidence of valve collapse while 58.9% had visible evidence of valve collapse on physical exam (*p* = 0.084) (Table 2).

**Table 2:** Summary of Patients with and without subjective evidence of nasal valve collapse on exam

Subjective NVC	N	NOSE (mean)	NOSE (SD)	Positive Cottle	Negative Cottle
Negative	65	14.78	3.9	39	26
Positive	76	11.79	4.1	56	20

The results of the ANOVA are presented in Table 3. Assessment of residuals versus fits appeared to be random and fit the model. There were 10 outliers in the model. The ANOVA was tested a second time with the outliers removed and there was no change in the results. There was no statistically significant difference in the NOSE score at 12 months between those patients with and without a positive pre-operative Cottle maneuver (*p* = 0.38, R-squared = 56.29%). The mean (95% C.I.) difference in NOSE score at 12 months between patients with a positive Cottle versus a negative Cottle was 0.18 (− 1.6 to 1.92).

**Table 3:** Summary of ANOVA for the primary outcome measure

Source of Variation	df	Sum of Squares	Mean Square	*f*-value	*p*-value
Baseline Score	1	1536.24	1536.24	147.72	< 0.001
Age	1	117.44	117.44	11.29	0.001
Cottle	1	7.96	7.96	0.77	0.383
Gender	1	1.58	1.58	0.15	0.697
Error	124	1289.54	10.4		
Total	128	3027.67			

*df* degrees of freedom

Performing the same ANOVA model with the presence of absence of visible valve collapse on exam did not reach significance (*p* = 0.27).

Of the 141 patients who completed the one-year follow-up, 14 did not meet the definition of surgical success. The causes of failure, as assessed by the primary author are listed in Table 4. In those patients that failed the surgery, the most common cause was a persistent caudal septal deviation (33%) followed by static nasal valve narrowing (27%). Dynamic collapse was the cause of only one surgical failure in our population. One patient failed due to nasal polyps that were not appreciated during the pre-operative evaluation.

**Table 4:** Causes of surgical failure

	Positive Cottle		Negative Cottle		All Patients	
Reason for Surgical Failure	Count	% Total	Count	% Total	Count	% Total
Caudal Septal Deviation	4	4.2	1	2.2	5	3.5
Narrow External Nasal Valve (Static)	3	3.2	1	2.2	4	2.8
Valve Collapse (Dynamic)	0	0.0	1	2.2	1	0.7
Perforation	0	0.0	1	2.2	1	0.7
Untreated allergy	2	2.1	0	0.0	2	1.4
Nasal Polyps	0	0.0	1	2.2	1	0.7
TOTAL	9	9.5	5	10.9	14	9.9

Results of the logistic regression failed to demonstrate the usefulness of the Cottle maneuver as a predictor of surgical failure in this population (*p* = 0.99). Specifically, a positive Cottle maneuver increased the odds of surgical failure by an odds ratio (95% C.I.) of 0.79 (0.22–2.8).

## Discussion

The diagnosis and treatment of nasal valve collapse in the context of a septal deviation can be challenging surgical and diagnostically. Published expert consensus states that there is no gold standard test to diagnose NVC [13]. Given the limited availability of objective measures to diagnose NVC, clinicians and surgeons utilize their history and physical exam. Understanding the efficacy of individual components of the physical exam and their relationship to nasal obstructive will provide a better understanding of the utility of these measures. This study provides evidence that as a single diagnostic measure, the Cottle maneuver has limited clinical utility in predicting which patients with nasal obstruction secondary to a septal deviation will fail a septoplasty and inferior turbinate reduction. One key assumption with this reasoning, however, is that those patients with a positive Cottle maneuver also have nasal valve collapse. Although this is not always the case, the Cottle maneuver is the most commonly utilized physical examination for excluding [14–21] and including [6] NVC in previous studies.

The results of this study provide evidence that many patients with a positive Cottle maneuver who undergo a septoplasty and turbinate reduction will demonstrate an equivalent improvement in their symptoms to those patients with a negative Cottle maneuver. The reduction in the NOSE score after a septoplasty in both positive and negative Cottle maneuver patients were similar to those in other published papers assessing patients without evidence of nasal valve collapse [16–18, 20, 40]. To our knowledge, there is no published study that provides evidence demonstrating the usefulness of nasal valve lateralization techniques or lateral nasal sidewall strengthening over a standard septoplasty with turbinate reduction in patients with mild to moderate valve collapse based on the Cottle maneuver. One important consideration, however, is that this study was an assessment of a single examination. It is likely that multiple factors predict the failure of a septoplasty, and several examinations considered together may be more appropriate than a single assessment using a single test. The goal of this study is to provide the basis of future studies assessing a multivariate assessment of nasal examinations of surgical outcomes.

Two recent systematic reviews evaluated the surgical treatment of NVC [6, 11]. None of the studies captured in the review compared the use of a septoplasty (with or without turbinate reduction) alone versus other methods of nasal valve repair (with or without a septoplasty) [6, 11]. In many of the reported studies a septoplasty was performed at the time of the nasal sidewall (nasal valve) surgical procedure; however, in no cases was this quantified or controlled as a confounding variable. A recent meta-analysis by Floyd et al. [10] noted that the NOSE score in patients with nasal obstruction due to NVC was significantly reduced following a functional rhinoplasty, with or without a cosmetic component. Although it was noted that a septoplasty is part of a functional rhinoplasty and performed for nearly all patients, the efficacy of this alone was not controlled nor accounted for in the statistical methods. In fact, the authors excluded any paper that included patients who had a septoplasty alone. In addition, in their series of 12 patients with a septal deviation and contralateral alar valve collapse, Schalek and Hahn reported that 11 of 12 patients reported significant improvement of nasal breathing following a septoplasty, without additional procedures to address the nasal valve [7]. Certainly, there is a role for functional rhinoplasty in many patients with nasal obstruction with NVC; however, an evidence-based approach to identify these patients, and to identify which patients will experience sufficient improvement with a septoplasty and inferior turbinate reduction alone, is currently lacking.

Considering surgical failure, dynamic NVC represented a surprisingly small number of surgical failures. The results of our study identify a caudal septal deviation followed by static nasal valve narrowing as the two most common causes of failure. Previous studies have noted that failure to recognize dynamic NVC pre-operatively is the most common cause of septoplasty failure [9, 32, 41, 42]. Chambers et al. (2015) [43] performed a retrospective assessment of patients who did not demonstrate clinical improvement after a septoplasty. Due to the lack of baseline population numbers, overall the rate of failure is not possible to calculate; however, the cause of failure appeared to be multifactorial. Further complicating the analysis of the results, the authors did not provide information on what tests were used to define specific causes failure causes.

Another unexpected finding in this study was that age was a significant covariate in the ANOVA model. When reviewing this outcome, although statistically significant, the relationship was weak and did not add any clinically meaningful predictive benefit. Prior studies failed to demonstrate any correlation between age and improvements after a septoplasty [22, 23], and therefore it is possible that the positive results in this study are due to being over-powered; it may in fact be a false positive outcome.

Although this study represents a high quality, prospective assessment of patients with nasal obstruction, there are some limitations. One limitation of the study design was that patients could not be randomized to undergo septoplasty and inferior turbinate reduction versus functional rhinoplasty and have post-operative results compared. Given the heterogeneity in both type and location of septal deviation, no specific data was collected with respect to the location of the septal deviation in our patients; this could have been of interest for a more detailed understanding of the etiology of individual patients’ NVC, and should occur in future studies. Similarly, we did not prospectively record other commonly utilized assessments of NVC pre-operatively, such as the modified Cottle maneuver. Specific findings in nasal endoscopy was not recorded in our pre-operative evaluation, apart from using it to rule out other causes of nasal obstruction. However, the authors of the AAO-HNS clinical consensus statement indicate that anterior rhinoscopy can be sufficient for an intra-nasal examination of the nasal valve [13] and therefore we did not include specific endoscopy information. We selected the NOSE score as our primary outcome measure, as the AAO-HNS clinical consensus statement indicates that a NOSE score is valid for the purpose of assessing the outcome of surgical interventions and that the NOSE scale was the most common outcome measure used in a systematic review of studies evaluation the surgical treatment of internal NVC [11, 13]. However, additional outcome measures such as visual analogue scales for nasal breathing could also have been of value, given that nasal breathing is subjective and not a dichotomous variable.

Another limitation of this study is that a single surgeon performed all assessments. Given a lack of a validated grading scheme, a general assessment of NVC is therefore subjective. Finally, biases can occur in assessment of surgical failure, and therefore a more robust and preferably blinded assessment would be optimal to validate these findings studies. However, we chose to use a patient centered definition of surgical failure, therefore limiting this bias.

The findings of this study have considerable applicability in terms of patient safety and health care resource utilization. Potential complications, as well as morbidity of more advanced surgical procedures are likely greater for a functional rhinoplasty than for a standard septoplasty, particularly if grafting is required from sites other than the nasal septum. With respect to health economics, in the practice of the primary author, a septoplasty and turbinate reduction can be performed rapidly, resulting in less time in the operating room and less post-operative care compared to more advanced functional rhinoplasty techniques specific for nasal valve collapse. The reduction in operative time, healing time and complications likely all contribute to lower health care costs, both direct and indirect. Future studies will be required to assess these questions.

In summary, this study demonstrated that there is no difference in patients with and without a positive Cottle maneuver when used as a single univariate assessment tool. In these patients, it should be used cautiously as a single outcome measure when predicting which patients may require nasal valve surgery and as an exclusion or inclusion criteria in research studies. However, it remains unclear if the test plays a role in a multivariable predictive model for detecting clinically relevant NVC. The results of this study could potentially influence practice, by encouraging clinicians to consider multiple factors when assessing the cause of nasal obstruction, as well as the need for advanced nasal surgery in addition to a septoplasty, and not simply relying on the Cottle maneuver as a dichotomous indicator of nasal valve collapse. Consequently, the accurate diagnoses of clinically relevant NVC requiring nasal sidewall repair continues to remains a challenge [44].

## Conclusion

The Cottle maneuver offers limited clinical utility to predict symptom improvement following septoplasty with inferior turbinate reduction in patients with nasal obstruction due to a septal deviation, with or without NVC. This study also suggests that a large proportion of patients with clinical evidence of NVC, based on the Cottle maneuver and physical examination, may not require advanced nasal valve procedures in addition to a septoplasty and turbinate reduction.

To date, there is no evidence-based outcome measure, or combination of outcome measures that predicts which patients will require more advanced nasal valve surgery. Certainly there remains a role for functional rhinoplasty to address the nasal valve; however, future studies are necessary to determine the variables that predict which patients are at a high risk of surgical failure, and to more accurately determine which patients with nasal obstruction and NVC require a functional rhinoplasty.

## Abbreviations

AAO-HNS: American Academy of Otolaryngology – Head and Neck Surgery
ANOVA: Analysis of variance
NOSE: Nasal obstruction symptom evaluation
NVC: Nasal valve collapse
OVCS: Ottawa Valve Collapse Scale
